# Observation of compositional domains within individual copper indium sulfide quantum dots†
†Electronic supplementary information (ESI) available. See DOI: 10.1039/C6NR03269A



**DOI:** 10.1039/c6nr03269a

**Published:** 2016-07-19

**Authors:** Andrew J. Harvie, Matthew Booth, Ruth L. Chantry, Nicole Hondow, Demie M. Kepaptsoglou, Quentin M. Ramasse, Stephen D. Evans, Kevin Critchley

**Affiliations:** a School of Physics and Astronomy , University of Leeds , Leeds LS2 9JT , UK . Email: k.critchley@leeds.ac.uk; b SuperSTEM , STFC Daresbury , Keckwick Lane , Warrington WA4 4AD , UK; c Institute for Materials Research , University of Leeds , Leeds LS2 9JT , UK

## Abstract

The origin of photoluminescence in copper indium sulfide (CIS) quantum dots (Qdots) has previously been ascribed to a donor–acceptor pair (DAP) recombination, with a crystal lattice defect implicated as the origin of the donor state. In this study, electron energy-loss spectroscopy (EELS) was used to observe defect-rich compositional domains within individual CIS Qdots, supporting a model of defect-state-mediated photoluminescence for these particles, and identifying them as an ideal model system for future study of lattice defects on Qdot properties.

## Abstract 2

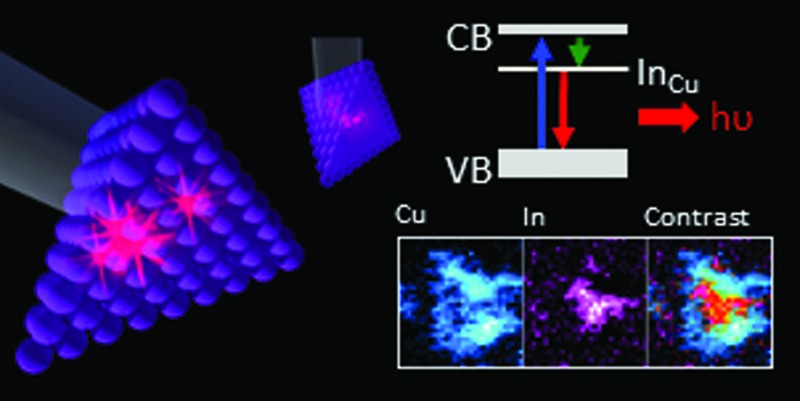
We report observation of highly-segregated compositional domains within CuInS_2_ quantum dots, showing the origin of their emission-mediating In_Cu_ defect.

Quantum dots (Qdots) are now widely used in many fields of research, from bioimaging,^1^ and display technology^2^ to solar energy harvesting;^3^ synthesis of many Qdot types with desirable properties is now often very simple. Recently, quantum dots are being integrated into commercial products, including display technologies.^4^ In spite of their widespread application, our understanding of the effects of crystal lattice defects on the electronic properties, including excited state dynamics and recombination mechanisms within Qdots is still poor. Copper indium sulfide (CIS) Qdots are particularly technologically important,^5,6^ since they offer emission from yellow to near infra-red. Furthermore, their low toxicity in comparison to their CdX (X = S, Te, or Se) counterparts^7^ makes them excellent candidates for use as fluorescent probes in biomedical research, and their wide absorption and high extinction coefficient mean they are viable as absorbing material for both light emitting diode and photovoltaic devices.^8,9^ Furthermore, the drive to reduce use of cadmium-based materials in electronics provides the need to use Cd-free Qdots.

CIS Qdots typically exhibit both a large Stokes shift and wide emission band in comparison with CdSe Qdots, for example.^9,10^ Recent literature has suggested that these phenomena are best explained by a model of donor–acceptor pair (DAP) recombination, implicating crystal lattice defects as the origin of the donor state;^7,11,12^ however, contradictory models are also present in the literature.^13–16^ Simulations of donor–acceptor pairs in Qdots show that donor and acceptor energy levels are dependent on the Qdot size.^17^ This explains the strong size dependence of the defect state-to-valence band emission observed in CIS Qdots.^18^ Kraatz *et al.* recently reported femtosecond pump–dump–probe spectroscopic data that indicate that the emissive transition for chalcopyrite CIS Qdots is a recombination event involving an intraband electronic state consistent with an In_Cu_ antisite defect.^10^

Studies comparing photoluminescent quantum yield (PLQY) for CIS Qdots with varying Cu : In ratios show that Cu-poor Qdots exhibit higher PLQY than equivalent stoichiometric (Cu : In : S – 1 : 1 : 2) chalcopyrite Qdots.^19,20^ This is attributed to an increase in the number of In_Cu_ and V_Cu_ (copper vacancies) in these Qdots; these defects are expected to have a lower formation energy in Cu-poor CIS.^21^

CIS Qdots that have been overcoated with a ZnS shell exhibit an enhanced PLQY compared with the core CIS Qdots, from approximately 4% to 65%.^22^ This enhancement is due to the passivation of surface trap states that are involved in non-radiative recombination of excitons. This improvement in the PLQY is also accompanied by a blue shift in the fluorescence emission, which has been attributed to cationic exchange between the CIS and ZnS lattices, most likely resulting in a semi-alloyed CIS/ZnS particle rather than a true “core–shell” structure with a well-defined boundary.^22^ This blue shift is often accompanied by a narrowing of the broad PL emission spectrum,^22^ which we attribute to a size-focussing of QDs *via* Ostwald ripening during the shell growth process.

To explain the nature and origin of the crystal defects that are related to emissive recombination in CIS Qdots, we perform here a Scanning Transmission Electron Microscopy-Electron Energy Loss Spectroscopy (STEM-EELS) study of stoichiometric CuInS_2_ (CIS) chalcopyrite Qdots. STEM-EELS is advantageous over methods such as EDX that has previously been used for chemical mapping in CIS nanoparticle samples^23^ due to its improved spatial resolution, especially in the *z*-direction.

The CIS and CIS/ZnS Qdots were synthesized as described previously, *via* a one-pot solvothermal method, which is posited as a viable batch-production strategy.^8,10,11,24^ The synthesis protocol is described in the ESI.† The properties of CIS nanoparticles are very sensitive to the synthesis methods chosen. For some solvothermal methods, changing the reaction temperature results in varying CIS structures, in some instances leading to mixtures of nanoparticles with different structures, including wurtzite (WZ) and zinc blende (ZB) existing within the same reaction mixture.^23^ Interestingly, the former of these materials displays a different mechanism of luminescence to chalcopyrite CIS Qdots; emission results from a transition involving an indium interstitial (In_i_) defect state.^25^ Considering the effect synthesis conditions can have on the structure, and therefore emission mechanism of CIS, the synthesis method described here was therefore chosen to preserve consistency with our previous work on pump–probe spectroscopy of the CIS/ZnS QDs, which assigned the In_Cu_ defect as that which is responsible for photoluminescence.

Survey images of large numbers of particles were obtained by high-angle annular dark field scanning transmission electron microscopy (HAADF-STEM). An example image is shown in Fig. 1; further images are included in the ESI.† Both CIS core and CIS/ZnS core–shell particles display a typically triangular projection, consistent with the expected tetrahedral morphology. For the core Qdots, the mean size (measured as the distance from one vertex of the triangular projection of the Qdot to the opposite side) was determined to be 2.4 ± 0.3 nm (*n* = 32), increasing to 2.7 ± 0.4 nm upon the addition of the ZnS “shell”. This relatively wide size distribution is fairly typical of Qdots synthesized by this or similar methods.^5^

**Fig. 1:**
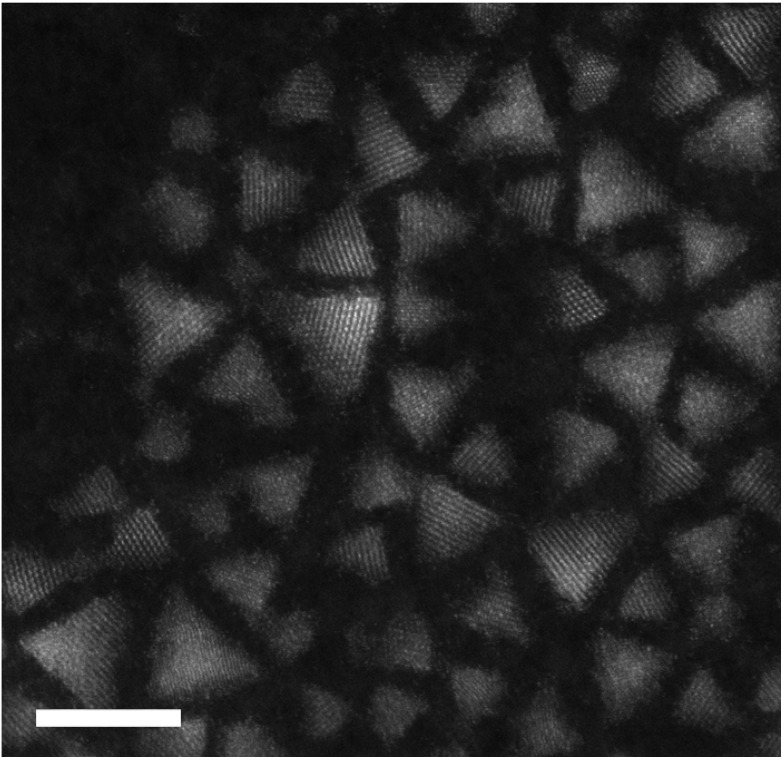
Survey image of the core–shell CIS/ZnS Qdots, displaying their tetrahedral morphology. Scale bar 5 nm.

PL emission and UV-Vis absorption data for the CIS and CIS/ZnS particles were acquired for colloidal samples dispersed in chloroform. Fig. 2 shows PL emission spectra for the core and core–shell samples, with intensities normalised to the value for absorption at 450 nm, which the chosen wavelength of excitation. The core particles display a broadband PL emission at 688 nm (FWHM 121 ± 1 nm), which is typical of CIS Qdots. Addition of the ZnS shell led to an 8.9-fold enhancement in the PL intensity, as well as a blue shift in the PL emission maximum by 43 nm (maximum 645 nm, FWHM 119 ± 1 nm).

**Fig. 2:**
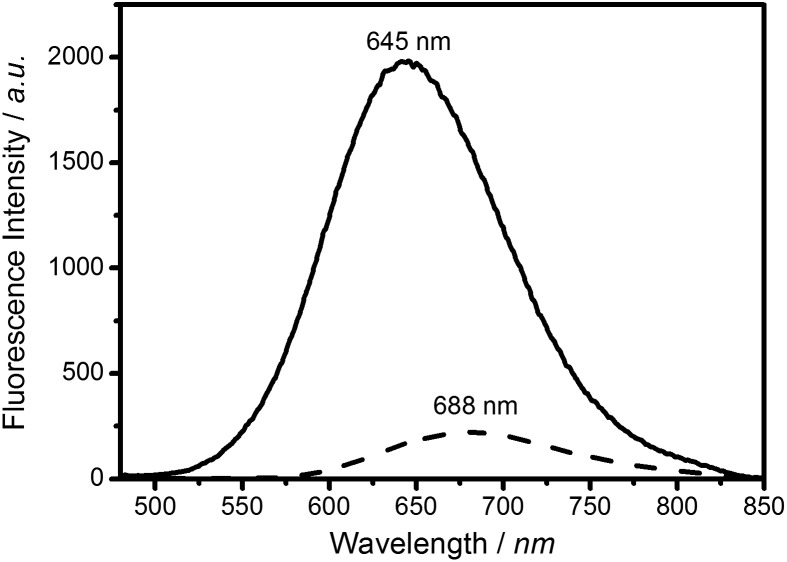
PL spectra of core CIS (dashed line) and core–shell CIS/ZnS (solid line) Qdots, with excitation at 450 nm. Addition of a ZnS “shell” results in a 43 nm blue shift of the PL peak, as well as an 8.9-fold increase in the photoluminescent intensity.

X-ray diffraction (XRD) was used to confirm the lattice structure of the core CIS Qdots, and is shown in the ESI.† The diffraction pattern was consistent with literature data for chalcopyrite CuInS_2_.^26^

X-ray photoelectron spectroscopy (XPS) of samples prepared by drop-casting a 5 μM solution of the core CuInS_2_ Qdots, in chloroform, onto gold-coated glass slides was performed to measure elemental ratios. Photoelectron peaks at binding energies of 932.3 and 445.8, 161.2 and 162.2 eV, corresponding to Cu 2p_3/2_, In 3d_5/2_ and an S 2p doublet respectively, were observed in good agreement with literature data for CIS nanoparticles.^27^ Detailed analysis gives the ratio of Cu : In : S to be 1.0 : 0.9 : 2.7. The excess of sulfur is attributed to the dodecanethiol (DDT) capping ligand. Importantly, the measured Cu : In ratio is stoichiometric within the expected instrumental uncertainty. Plots of the Cu 2p_3/2_ and In 3d_5/2_ used to quantify the Cu : In ratio are shown in the ESI.†

EELS maps of Cu and In were obtained for samples of CIS core and CIS/ZnS core/shell Qdots. All EELS maps were obtained using a Nion UltraSTEM™ microscope. Maps were obtained of Qdots in areas with a relatively low surface number density; we found that for longer acquisition times, the quality of EELS maps for particles with many close neighbours decreased. Depending on the resolution used, the acquisition time for each map was between 7–15 minutes. Each pixel consists of an EEL spectrum for the corresponding location in the image plane. For each spectrum, Cu and In edges were located, and after subtraction of the background signal using a power-law fit immediately prior to the relevant edge, the intensities were extracted to produce maps showing the spatial distribution of these elements. Some representative results are summarized in Fig. 3. It is important to note that these maps are not quantitative; for presentation the contrast in each map has been normalized to unity.

**Fig. 3:**
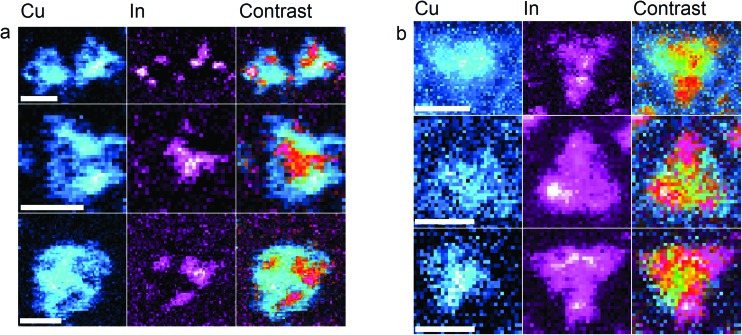
Elemental maps of Cu and In for CIS core (a) and CIS/ZnS “core–shell” (b) quantum dots. The column labelled “Contrast” is a subtractive RGB difference overlay of the respective Cu and In maps. On each Qdot, areas of high In signal correspond with a low Cu signal and *vice versa*, showing segregation of Cu and In within particles. It is important to note that these maps are not quantitative; for presentation the contrast in each map has been normalized to unity. Scale bars 2 nm.

As is shown in Fig. 3, STEM-EELS elemental maps of the particles systematically show separation of copper and indium within individual particles, giving both In-rich and In-poor areas, which spatially correspond to Cu-poor and Cu-rich, respectively. This is observed in both CIS core and CIS/ZnS core/shell particles. In general, indium-rich areas are distributed seemingly randomly within the Qdot, and are between 4–8 Å in size. For our samples, the number of In-rich segregated areas per particle ranged between 1 and 4. Evidence of these segregated areas immediately suggests an origin of the In_Cu_ antisite defect suggested from spectroscopic data; In-rich/Cu-poor regions are expected to have a number of In_Cu_ displacements in stoichiometric CIS if the chalcopyrite lattice structure is preserved, as evidenced by XRD. Furthermore, studies of formation energies in CIS and the analogous chalcopyrite copper indium selenide (CISe) suggest a low formation energy for these In_Cu_ defects, especially when present as part of a In_Cu_ + 2V_Cu_ defect pair.^28,29^ Presence of the In_Cu_ + 2V_Cu_ defect system is also known to promote formation of Cu_In_ + In_Cu_ defect pairs, resulting in large “clusters” of defects within the lattice.^30^ It is worth noting that the formation energy of In_Cu_ is expected to be lowered in copper-poor CIS, concurrent with the observation of increased quantum yield in these off-stoichiometric Qdots.^21^ In fact, the large tolerance to off-stoichiometry displayed by CIS in the Cu-poor regime has been explained by the stability of the In_Cu_ + 2V_Cu_ pair.^31^

Interestingly, for the CIS/ZnS “core–shell” particles, the degree of segregation in some particles appears smaller than that of the core CIS Qdots. This may be due to an annealing process that occurs during the shell growth step, as well as the internal rearrangement of the lattice as the aforementioned cationic exchange with ZnS takes place.

Our study shows compositional heterogeneity alongside the preservation of the chalcopyrite structure, consistent with the presence of the theorized In_Cu_ antisite defect. Compositional heterogeneity within single crystal CIS of this or similar size has until now not been observed.

This compositional heterogeneity within particles will lead to a wide variance in environments for the donor-state In_Cu_ defects; the length scale across which composition varies (as large as 1 nm in some particles) is frequently larger than that of the size of a single In_Cu_ + 2V_Cu_ defect pair. It has previously been shown that the energy level of an impurity or defect-related electronic state is strongly dependent on its environment – in this case this is not only the location of the defect within the particle, but the local composition; the band gap of CuInS_2_ has been shown to be closely related to the Cu : In ratio, and the energy of these defect states is dependent on the local band gap.^9,32,33^ Compositional heterogeneity therefore leads to heterogeneity in both band gap and defect state energy within individual particles. This effective broadening of features of the band structure in turn therefore leads to the broad PL emission characteristic of CIS Qdots.

The optical characteristics of CIS Qdots are in agreement with the model of In_Cu_-defect mediated DAP emission, with the observed compositional heterogeneity identified as the origin of these defects. Although many synthesis routes for these Qdots are present in the literature, and it is not possible to confirm that these will all yield equivalent internal compositional distribution, the characteristic wide PL and large Stokes shift displayed by chalcopyrite Qdots from many synthesis routes does suggest internal compositional heterogeneity is an inherent feature of chalcopyrite CIS Qdots.^5^

In conclusion, we have used STEM-EELS mapping to image elemental distribution of copper and indium within solvothermally synthesized chalcopyrite CIS quantum dots. All Qdots investigated displayed a degree of separation of Cu and In, without any apparent discontinuity in the chalcopyrite lattice structure. This is a new observation for chalcopyrite CIS quantum dots. The observed separation of Cu and In is consistent with a large population of In_Cu_ defects, which support intraband electronic states involved in radiative recombination of excitons, leading to the characteristic photophysical properties of CIS quantum dots.

This new understanding of the presence of natural intrinsic defects within CIS Qdots identifies them as an ideal platform for further study of the control and effects of defects on Qdot properties; it has already been shown that the defect-related emission can be tuned during introduction of zinc *via* exchange during the shell growth step.^22^ The segregated, defect-rich nature of these particles can be considered analogous to that of doped semiconductor Qdots, providing a simple model platform for their study.^34^ Engineering of other intrinsic and extrinsic defects can therefore lead to further developments in customising Qdots for emerging technologies.

SuperSTEM is the EPSRC National Facility for Aberration-Corrected Scanning Transmission Electron Microscopy. AJH thanks AstraZeneca for contributions to funding. KC thanks the MRC and EPSRC for financial support (grant references MR/K015613/1, EP/K023845/1). The data associated with this paper are openly available from the University of Leeds Data Repository http://doi.org/10.5518/89.
