# Supplementary material for: Observation of compositional domains within individual copper indium sulfide quantum dots
Source: Nanoscale. 2016 Jul 19;8(36):16157–61. doi: 10.1039/c6nr03269a (PMC5048650; doi:10.1039/c6nr03269a)
Supplement: Supplementary file 1 [file NR-008-C6NR03269A-s001.pdf]

Electronic Supplementary Material (ESI) for Nanoscale.
This journal is © The Royal Society of Chemistry 2016

Observation of Compositional Domains Within Individual Copper Indium Sulfide Quantum Dots - Supporting Information

Andrew J. Harvie[1], Matthew Booth[1], Nicole Hondow[2], Ruth L. Chantry[3], Demie M. Kepaptsoglou[3], Quentin M. Ramasse[3], Stephen D. Evans[1] and Kevin Critchley*[1]

[1]School of Physics and Astronomy, University of Leeds, Leeds LS2 9JT, United Kingdom
[2]Institute for Materials Research, University of Leeds, Leeds LS2 9JT, United Kingdom
[3]SuperSTEM Laboratory, STFC Daresbury, Keckwick Lane, Warrington WA4 4AD, United Kingdom

Quantum Dot Synthesis

For the synthesis of the core CIS quantum dots, copper iodide (0.25 mmol) and indium acetate (0.25 mmol) were added to a mixture of dodecanethiol (DDT)(4ml) and octadecene (ODE)(6ml) under a protective argon atmosphere, before stirring at room temperature for one hour to remove dissolved oxygen. The solution was then refluxed at 120°C where all solid material was seen to dissolve, then at 180°C for 20 minutes, at which point it became a deep red colour, indicating the formation and growth of nanoparticles. The growth was then quenched by rapidly cooling the reaction vessel by immersion in water at room temperature. Half of the reaction volume was then removed and kept. For the addition of a ZnS shell to form CIS/ZnS core-shell QDs, 0.125 mmol zinc stearate was added in 4ml ODE to the remaining core particles, before refluxing at 200°C for 60 minutes. The resulting core and core-shell quantum dots were then cleaned by dialysis in chloroform using regenerated cellulose membranes, before storage in an oxygen-free environment. All synthesis and cleaning steps were performed under a protective N_2 or Ar atmosphere.

*k.critchley@leeds.ac.uk

Sample Preparation

Samples were prepared for the STEM and STEM-EELS measurements by drop-casting a 5μM solution onto the ultra-thin carbon TEM grid. After evaporation of the solvent, the samples were baked overnight under vacuum at 100°C to remove any excess organic material before loading into the microscope. Samples that were not baked were also prepared and imaged; it was confirmed that the baking did not affect the shape or chemistry of the particles, although reduction in contamination of the baked sample made particle acquisition and mapping easier. Measurements were taken with an accelerating voltage of 60kV; higher beam energies caused damage to the particles in-situ.
For the XPS and XRD measurements, samples were prepared by drop casting concentrated Qdot solutions in chloroform onto a gold surface and a silicon zero diffraction plate, respectively, before allowing the solvent to evaporate, leaving a dry powder.

Characterisation data

Imaging and Particle Sizing

Supplementary figure shows HAADF-STEM images used to obtain sizing information for the core CIS and core-shell CIS/ZnS Qdots. For these particles, "size" is defined as the distance between one vertex and the opposite side of the triangular projection of the image. Only particles where this triangular projection was clear were selected for sizing. All images for this study were acquired on the Nion UltraSTEM™instrument, with the exception of the initial images of the core particles, which were obtained using a FEI Tecnai TF20 microscope. For the STEM-EELS measurements, a beam energy of 60kV was used; higher beam energies caused damage to the particles in-situ.

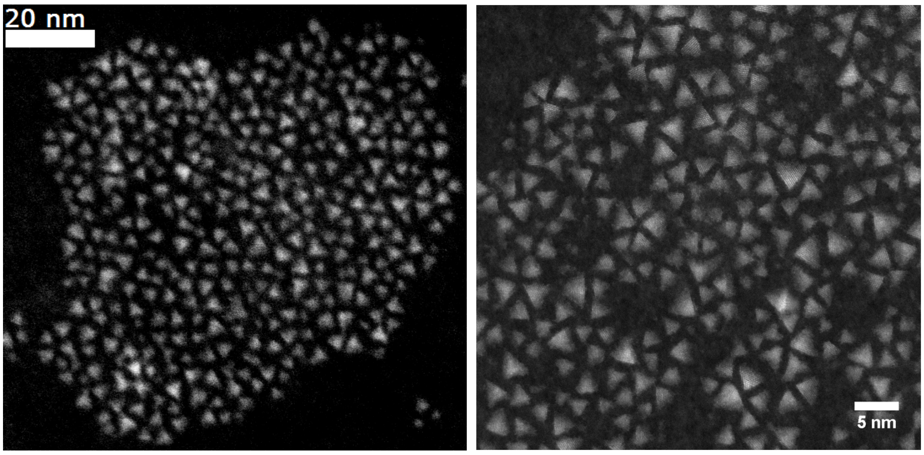

Supplementary Figure 1: HAADF-STEM images of core CIS (left) and core-shell CIS/ZnS Qdots (right), displaying the triangular projections used to size the particles.

Supplementary Figure 2 shows histograms of measured particle size for the CIS core and CIS/ZnS core-shell Qdots immediately post synthesis. The average size was seen to increase from 2.4 ± 0.3 nm to 2.7 ± 0.4 nm after the shell growth step, showing addition of shell material.

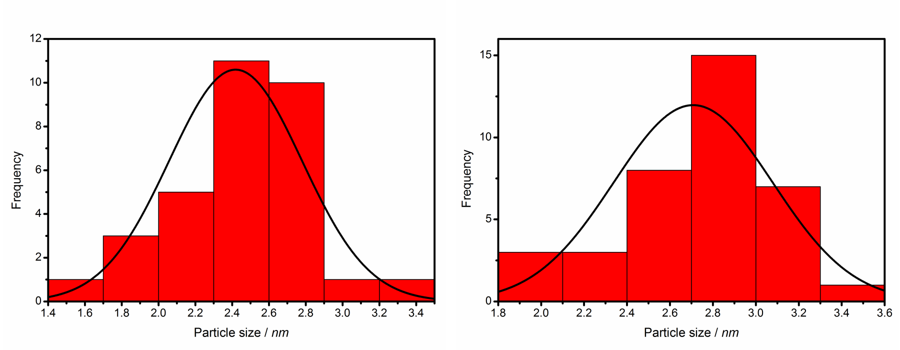

Supplementary Figure 2: Histograms of particle sizes as measured by TEM for core (left, n=32) and core-shell (right, n=37) particles as measured by STEM. The overlaid curves are Gaussian fits.

XRD Analysis

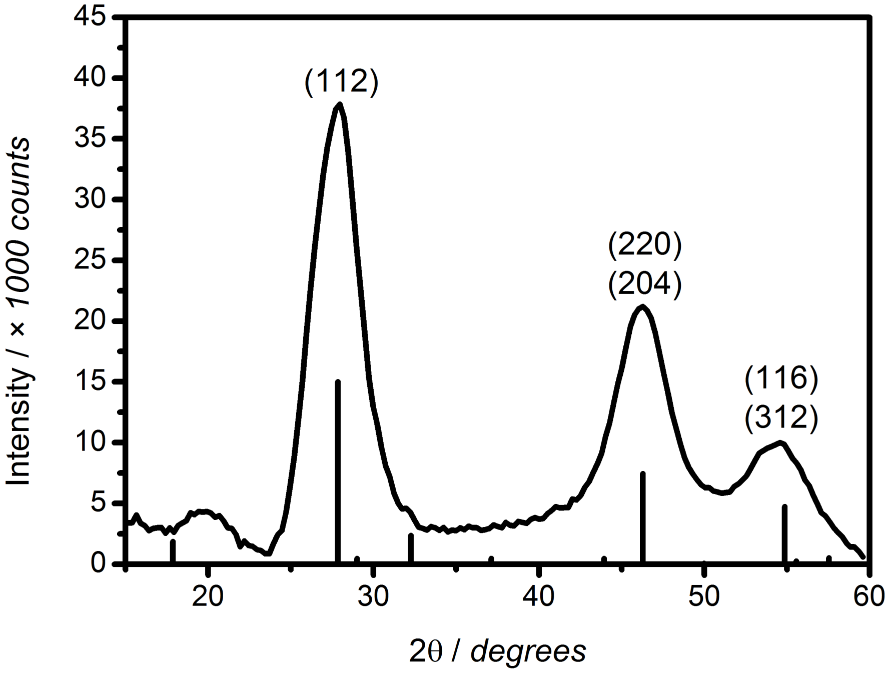

Supplementary Figure 3: Powder X-ray diffraction(XRD) pattern of dried sample of CIS Qdots. The spectrum correlates well with literature data for chalcopyrite $CuInS_2$.[1] (ICDD 04-005-5202)

Samples of the core particles were prepared for X-ray diffraction analysis as described above. Angle scans were taken over 25 minutes between 15° and 60°. The powder diffraction pattern was matched with chalcopyrite CIS. [1] (ICDD 04-005-5202)

Stoichiometry Quantification Using XPS

Samples of core CIS Qdots were prepared for XPS analysis as described previously, to check for Cu/In ratio. Photoelectron peaks for In3d$_{\frac{5}{2}}$, Cu2p$_{\frac{3}{2}}$ and S2p were fitted and used for determination of particle stoichiometry using Thermo Scientific ™Avantage software with Scofield's relative sensitivity factors.

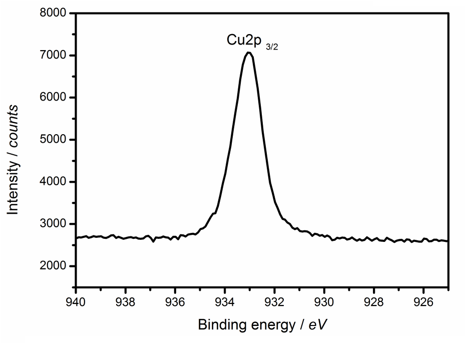

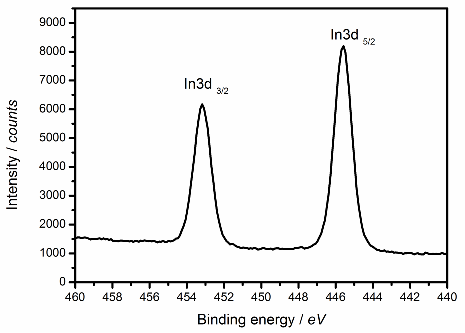

Supplementary Figure 4: X-ray photoelectron peaks for Cu2p$_{\frac{3}{2}}$ (left) and In3d (right) used to quantify Cu/In ratio in core CIS Qdots.

Data availability

The data associated with this paper are openly available from the University of Leeds Data Repository http://doi.org/10.5518/89.

References

[1] C. Sombuthawee, S. Bonsall and F. Hummel, *J. Solid State Chem.*, 1978, **25**, 391–399.
